# Statistical characteristics of tonal harmony: A corpus study of Beethoven’s string quartets

**DOI:** 10.1371/journal.pone.0217242

**Published:** 2019-06-06

**Authors:** Fabian C. Moss, Markus Neuwirth, Daniel Harasim, Martin Rohrmeier

**Affiliations:** Digital and Cognitive Musicology Lab, Digital Humanities Institute, College of Humanities, École Polytechnique Fédérale de Lausanne, Lausanne, 1015 Vaud, Switzerland; ISEP Instituto Superior de Engenharia do Porto, PORTUGAL

## Abstract

Tonal harmony is one of the central organization systems of Western music. This article characterizes the statistical foundations of tonal harmony based on the computational analysis of expert annotations in a large corpus. Using resampling methods, this study shows that 1) the rank-frequency distribution of chords resembles a power law, i.e. few chords govern a large proportion of the data; 2) chord transitions are referential and chord predictability is significantly affected by distinguished chord features; 3) tonal harmony conveys directedness in time; and 4) tonal harmony operates differently at the hierarchical levels of chords and keys. These results serve to characterize tonal harmony on empirical grounds and advance the methodological state-of-the-art in digital musicology.

## Introduction

One of the core questions in music research concerns the structural regularities within and across historical styles and cultures. In the field of music theory, manifold attempts have been made to characterize these structures and their underlying rule systems, from antiquity up to the present [1–9]. For the understanding of Western music, *tonal harmony* is perhaps the most central concept, setting it apart from other traditions in the world [10, 11]. However, previous music theoretical approaches addressing tonal harmony suffer from a lack of empirical foundation. When making general statements, they tend to rely on qualitative descriptions based on a small number of examples [8, 12–14], rather than on quantifiable and testable hypotheses.

In an attempt to fill this lacuna, recent initiatives in the field of computational musicology have adopted a *distant reading/listening* approach [15, 16] by exploring the structural properties of tonal harmony and applying statistical methods to various digital datasets [17–23]. Naturally, the empirical study of sophisticated concepts related to tonal harmony depends on tractable representations of musical structure. However, since symbolic representations of musical pieces are scarce, large-scale analyses have been hindered due to the lack of large symbolic corpora.

The goal of this study is to characterize the essential features (dimensions) of tonal harmony on quantitative grounds by applying statistical methods to a recently published dataset, the *Annotated Beethoven Corpus* (ABC) [24]. The ABC is an extensive, expert-curated corpus with approximately 28,000 structured chord labels added to digital scores of Beethoven’s string quartets. If performed, the whole set of string quartets would have a duration of approximately eight hours.

### The notion of tonal harmony

Tonal harmony is commonly associated with the historical period between the middle of the 17th and the second half of the 19th century, the so-called *common-practice period* [14, 25]. The organization principles of tonal harmony are still prevalent in contemporary Pop and film music [26–30]. Despite notable differences in the conception of tonal harmony, many theoretical treatises share the focus on a small number of central features [10, 11], which are here subsumed under the dimensions of *centricity*, *referentiality*, *directedness*, and *hierarchy*. The concept thus entails specific organization principles for, and not the mere use of, tones in musical compositions.

The first dimension, *centricity*, relates to the structure of the harmonic lexicon and states that tonal harmony is governed by a few central chords. The most common notational and analytical system for chords uses Roman numeral symbols to refer to the root of a chord. It further proposes certain operations on chords, such as “inversion” (permutation of the chord notes), “suspension” (temporarily replacing chord notes by neighboring notes), or “addition of non-chord notes.” This system has recently been formalized [24] and is used for the analyses in our study. See section “Annotation standard” for a detailed description of this formalization.

The second dimension of tonal harmony is *referentiality*. Chords do not occur in random order but are governed by syntactical rules [31–33]. This involves specific chords to act as points of reference, towards which other chords are oriented. Referentiality occurs on all levels of structure, involving global relationships between a main key and subordinate keys as well as local relationships between chords within a given key [6]. Within keys, the main point of reference is called the *tonic* and notated with the Roman numerals I and i for the major and the minor mode, respectively. The tonic is assumed to be connected to *dominant* and *subdominant* sonorities (V and IV, respectively, for the major mode). Dominant sonorities, in turn, are said to be prepared by chords taken from the class of pre-dominant sonorities (e.g. ii, IV, or V/V in major), thus forming lower-level points of reference. Referentiality can be approximated by frequency: chords that are frequently targeted by other chords also occur more frequently in general. Note that referentiality is, however, in principle independent of temporal order, and thus cannot fully account for the sense of directedness characterizing tonal harmony.

*Directedness*, the third dimension of the present conceptualization of tonal harmony, predicts a preference for asymmetric chord progressions. A chord transition A → B is *asymmetrical* if chord A proceeds more often to chord B than vice versa [18]. This has cognitive implications, as the statistical regularities of chord transitions in tonal music arguably impact on the formation of listening expectations through implicit learning [17, 34, 35]. This suggests that chord progressions are organized to convey direction in time and thereby support the build-up of expectation and release. Transitions between chords are commonly classified into two distinct categories, authentic and plagal transitions, depending on the size and direction of the interval between the involved chordal roots. The descending fifth is a prototypical example of an authentic transition; it is generally identified as central for tonal harmony [7, 8, 36, 37] as opposed to other Western musical styles [38], especially when its goal is the tonic (V → I, or V → i). Further, the presence of chord types with certain features contributes to both referentiality and directedness. This applies, for instance, to dissonant chords such as seventh chords and suspensions, as they create specific expectations of the following chords [8, 17].

A fourth dimension of tonal harmony is *hierarchy* [6, 39, 40]. On the bottom level, this involves chords, their hierarchical relationships, and their subsumption under a given key; on the top level, it involves local keys, their hierarchical nesting, and their relationship to the global key. Whether the same principles operate on all levels (the *hierarchical uniformity* hypothesis) is an open issue [41, 42] that will be discussed below.

## Dataset

The corpus for this study is the ABC [24] which consists of 28,095 chord symbols (*chord tokens*) in total (1,131 unique *chord types*). Its annotation system exceeds those of most previous datasets compiled for computational music analysis, as it is strictly formalized and is able to express a broader variety of features. At the same time, it preserves essential components of a traditional representation schemes, namely Roman numeral symbols. The ABC was chosen because of the central role of Beethoven in music history and his influence on subsequent musical developments [43]. The string quartets were composed in a range of ca. 25 years (1800–1826), covering the composer’s middle and late productive phases, and hence the high Classical as well as the early Romantic eras. They comprise 70 movements in total, of which 42 (60%) are in the major and 28 (40%) are in the minor mode. The ABC contains 929 segments defined by local key regions, 357 in major and 572 in minor. These two modes have been found to differ with respect to their distributional statistics [17, 44]. Since global key regions, i.e. movements, are in fact mixtures of local keys, one can assume that local keys are more homogeneous with respect to harmony. Therefore, the subsequent analyses distinguish between major and minor and compare them on the segment level.

### Annotation standard

A full description of the annotation standard used in the ABC is given in [24]. Here, we present a short summary describing its main components that are necessary for understanding our results. All chord symbols start with a *root* that determines the relation of a chord to the local key. Major chords are specified by uppercase Roman numerals, while minor chords are specified by lowercase numerals (e.g. I, V, iii and vii).

**Fig 1:**
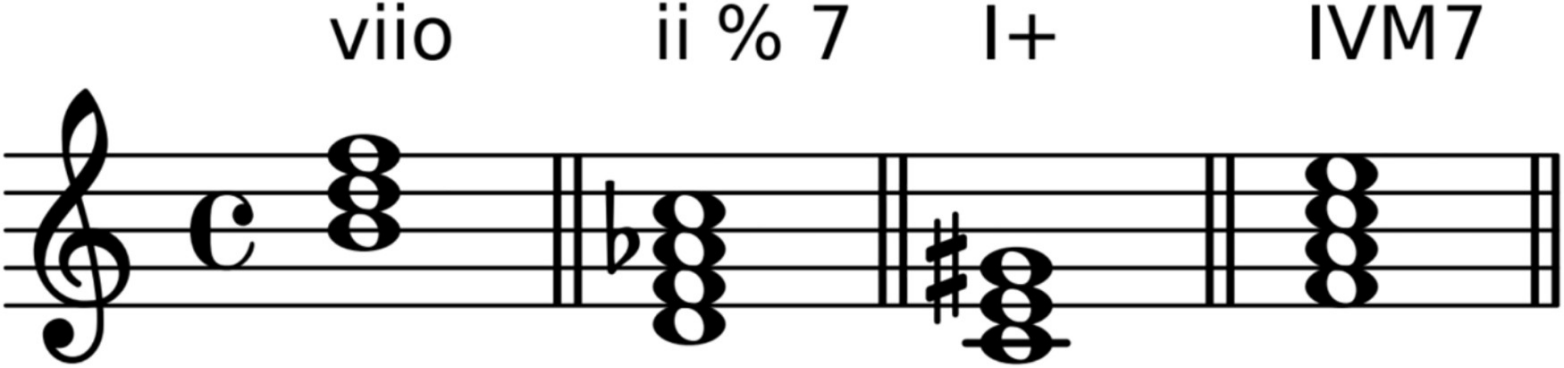
Chord forms. Examples of diminished, half-diminished, augmented, and major seventh chords with notation according to the ABC standard.

Apart from major and minor chords, the annotation standard distinguishes four more *chord forms*, namely diminished, half-diminished, augmented, and major seventh, which are encoded by the symbols o, %, +, and M7, respectively. Fig 1 shows examples of such chords.

**Fig 2:**
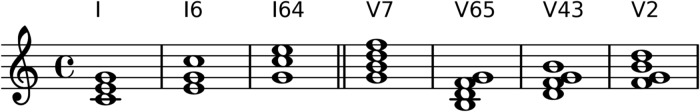
Chord inversions. Examples 1–3 illustrate a root-position chord and its first and second inversion; examples 4–7 show a seventh chord in root position and its first, second, and third inversion.

*Chord inversions*, the permutation of chord notes, are indicated by the symbols 6 and 64 for the first and second inversion of triads, and 7, 65, 43, 2, for sevenths chords in root position, first, second, or third inversion. The Arabic numbers show the intervallic distances between the bass (the lowest voice) and the upper voices. Fig 2 exemplifies root-position sonorities and their possible inversions.

**Fig 3:**
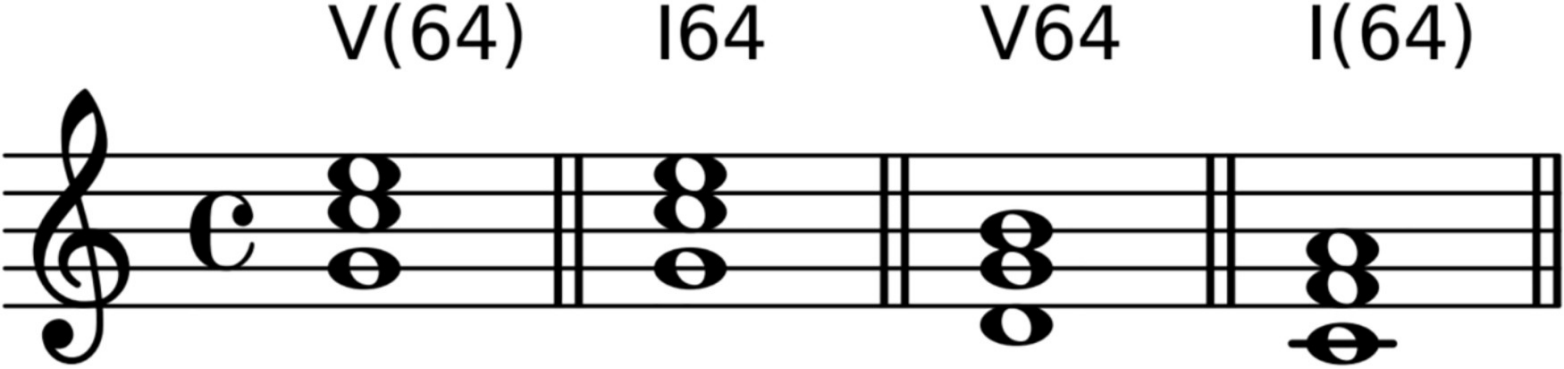
Chord suspensions and inversions. Examples of chord inversions (no brackets) and suspensions (brackets).

*Suspended and added notes* are indicated by bracketed Arabic numbers, which denote the intervallic distances of the upper voices to the chordal root; the same is true for added notes, which, however, are preceded by a +. Fig 3 shows the difference between suspended and inverted chords. Note that the first two chords are identical with respect to their pitch content and arrangement, but their harmonic function is interpreted differently due to the context (not shown). The first chord acts as a dominant with 64-suspension, and the second chord is an inversion of a tonic triad (both in C major).

**Fig 4:**
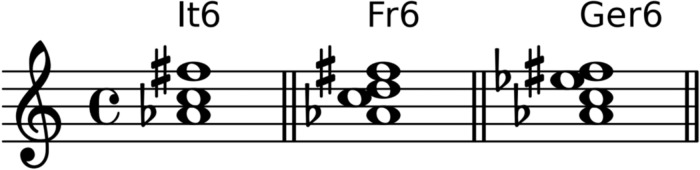
Augmented sixth chords. The Italian, French, and German augmented sixth chords in the key of C major.

*Applied chord*, i.e. chords that prepare or imply another chord (e.g. V7/vi, iv/bVI) are expressed by a slash symbol. Special chords are the three variants of augmented sixth chords [8], namely It6, Fr6, and Ger6. Fig 4 shows these chords as they would occur in the context of C major.

## Centricity: Structure of the chord lexicon

An important aspect for the characterization of tonal harmony is the used chord lexicon which we analyze with an *n*-gram model as is standard in Natural Language Processing (NLP) [45, 46]. Applied to chords, the underlying assumption of this model is that the probability of a chord *c*_*i*_ from a sequence *c*_1_, …, *c*_*i*−1_, *c*_*i*_ does not depend on its full history, but can be approximated only by the *n* − 1 chords immediately preceding it,

$$\begin{array}{c} p\left(c_{i}|c_{1},...,c_{i-1}\right)\approx p\left(c_{i}|c_{i-n+1},...,c_{i-1}\right), \end{array}$$ (1)

also called the *Markov assumption*. This study employs a unigram model (*n* = 1) to investigate structural regularities in the chord lexicon, and a bigram model (*n* = 2) for the analysis of chord transitions.

The corpus contains 16,544 chord tokens (794 chord types) in the major segments and 11,551 chord tokens (731 chord types) in the minor segments. Thus, the number of chord types for both modes are approximately equal. However, since the sets of chord types for both modes are not mutually exclusive, the number of chord types for all segments is not equal to the sum of types for the two modes. The unigram model accounts for the relative frequencies of chord types without accounting for the internal structure of the chords (e.g., V7 and V65 are different chords in the same way as I and V7 are different chords). This aspect is later remedied in the bigram model.

**Fig 5:**
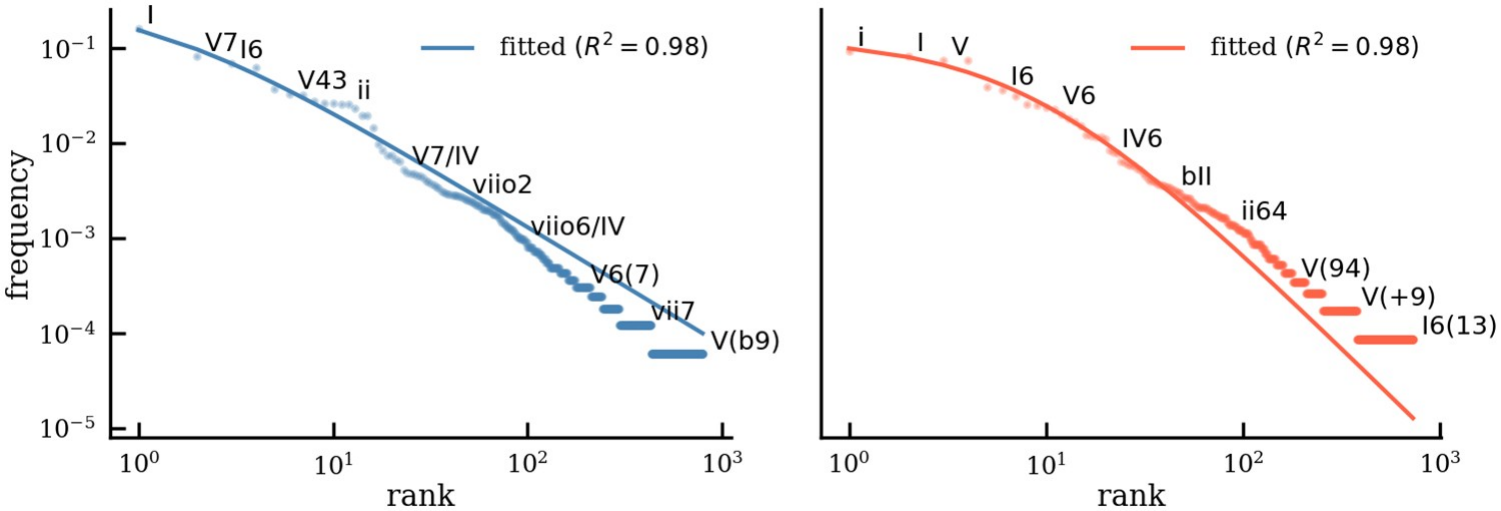
Chord frequency distribution. Rank vs. frequency plot of chords in major (left, blue) and minor (right, red) reveals an underlying power law. The solid line shows the best fit of a Zipf-Mandelbrot distribution as determined by the coefficient of determination *R*^2^.

The pattern of the rank-frequency distribution of chords resembles a power law, a well-known behavior of corpora in computational musicology as well as linguistics and other domains [18, 47–49]. Given the frequency rank *r* of chords, its frequency *f* can be approximated by a Zipf-Mandelbrot curve $\hat{f}$,

$$\begin{array}{c} \hat{f}\left(r\right)=\frac{\alpha }{{\left(\beta +r\right)}^{\gamma }}, \end{array}$$ (2)

for suitable parameters *α*, *β*, and *γ* [50, 51]. Fig 5 shows rank vs. frequency plots for all chord types in major (left, blue) and minor segments (right, red). The solid line is the fitted curve. The optimal curve parameters were determined via non-linear least squares. Accuracy of the fit is measured by the coefficient of determination *R*^2^ = 1 − (*SS*_*res*_/*SS*_*tot*_), where $SS_{res}=\sum_{r}{\left(f\left(r\right)-\hat{f}\left(r\right)\right)}^{2}$ and $SS_{tot}=\sum_{r}{\left(f\left(r\right)-\bar{f}\right)}^{2}$ are the residual sum of squares and the total sum of squares, respectively, *f*(*r*) is the empirical frequency of a chord type with rank *r*, and $\bar{f}$ is the mean of the empirical frequencies. The coefficient of determination is a suitable measure for the appropriateness of the curve fit because of its relation to the ratio of unexplained variance. On the other hand, this coefficient is influenced most by the top chords, as can be seen by the rather poor fit of the tail of the two distributions in Fig 5.

**Fig 6:**
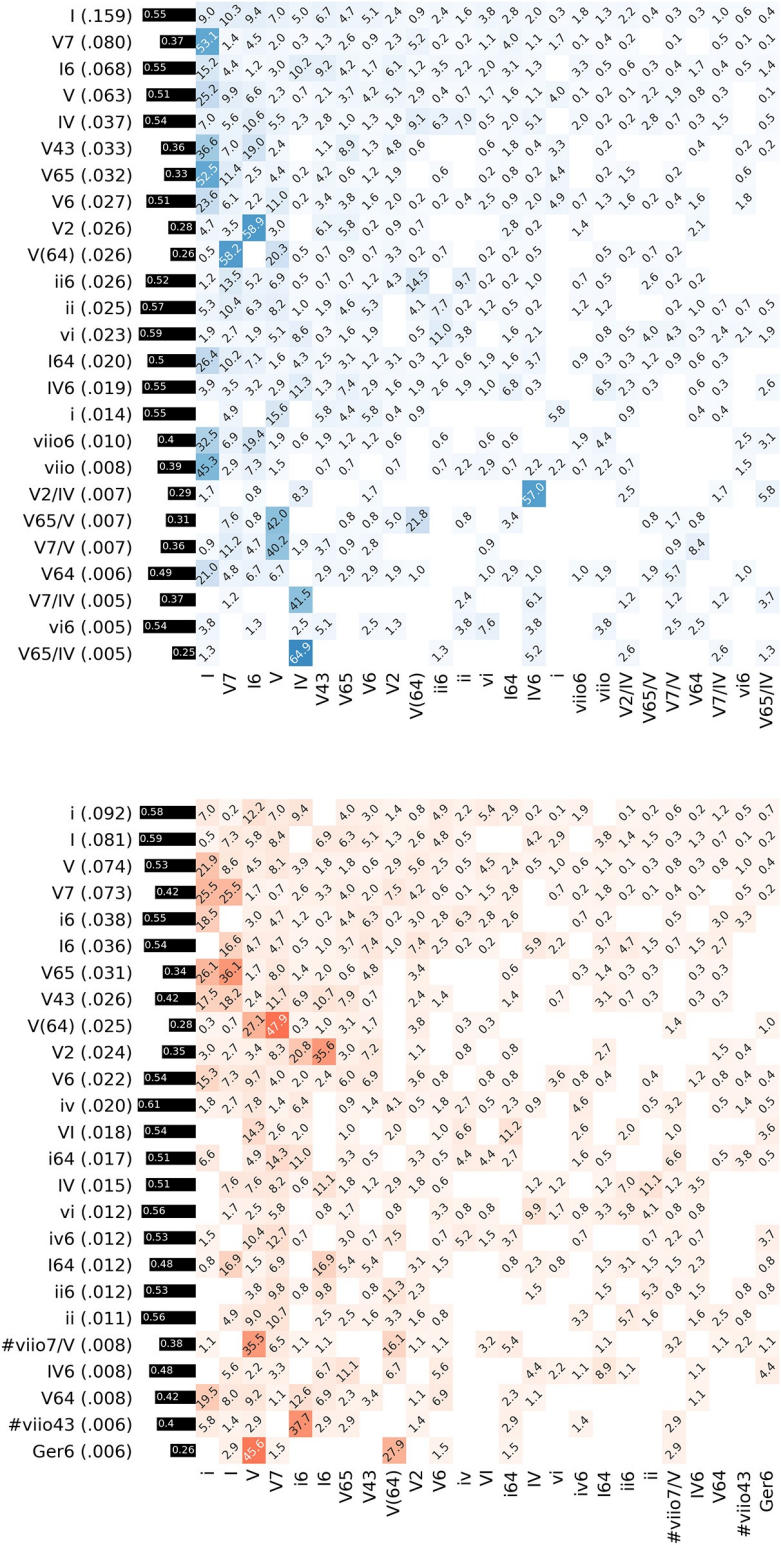
Chord transition probabilities. The rows in these heatmaps show the transition probabilities between the 25 top ranking chords in major (left, blue) and minor segments (right, red) as percentages. The black bars show the normalized conditional entropies over these distributions.

While one should be cautious not to over-interpret the shape of the distribution [52] and considering that further statistical tests would be required to determine whether they are truly Zipfian, the unigram distribution of chords does reveal the elevated roles of certain chords. The top 25 chords in both modes and their relative frequencies (in parentheses) are displayed as row labels of the heatmaps in Fig 6. The tonics, I in major and i in minor, are by far the most common chords. The V chord and its variants, such as V7, V43, V65, and V(64), govern most of the top ranks. Chords with roots I and chords with root V cover 26.1% and 40.5% of all chords in major, respectively. They together constitute more than 2/3 of the total probability mass. In minor, these quantities are 16% for chords with root i and 38.3% for chords with root V (in sum more than 50%). Chords with other roots such as IV and ii are much less frequent, irrespective of their forms of appearance (e.g., root-position or inversion). Chords such as iii or III are particularly rare and do not appear among the top 25 chords in either mode. This shows that, although the chord lexicon is of considerable size, only a small fraction of chords suffices to govern the main proportion of the data. In particular, I, i, and V chords account for more than 60% of all chords in major, and more than 50% of all chords in minor, clearly demonstrating their primacy in tonal music which reflects the principle of centricity. The chord distribution also sets tonal harmony apart from Rock/Pop tonality which likewise evinces centricity, but favors IV chords over V chords [38].

## Referentiality: Chord transitions

Regularities in the transitions between chords (chord bigrams) are important factors for the statistical description of a given musical style. Fig 6 displays statistics of chords and chord transitions in the major segments (top, blue) and in the minor segments (bottom, red) as heatmaps. The chord symbols on both axes correspond to the 25 most frequent chords in the respective modes. The heatmaps show the transition frequencies between the most frequent chords as percentages. The transition frequencies from a concrete chord symbol a (rows) to another concrete chord symbol b (columns) are used to estimate the transition probabilities *p*(a → b). Most of the top 25 chords clearly favor one particular continuation. In major, the dominants V7, V, V43, V65, V6, and V64 all proceed most frequently to I, as does viio, whereas V2 proceeds most often to I6, as is expected because of the voice-leading connection between the bass notes. The suspended V(64) chord resolves almost 80% of the time to either V7 or V. The applied chords V7/V, V65/V, V7/IV, V65/IV, and V2/IV all most likely proceed to their implied chords V and IV, respectively. In minor, the pattern is similar but slightly distorted by the high proportion of I chords in minor segments. The dominant chords V and V7 most commonly proceed to i or I. The inversions V65 and V43 even slightly favor continuations to I over i. Like in major, the V(64) chord in minor resolves into V7 or V. Moreover, the Ger6 chord, rank 25 in minor, proceeds most commonly to V, which is both predicted by voice-leading rules and its predominant function. V2 proceeds to either I6 or i6 which likewise reflects voice-leading conventions.

These results support the hypothesis that certain chord features, such as inversions and suspensions, have an impact on how well the continuation can be predicted. The certainty in prediction can be measured by the entropy of the transition probabilities. More generally, given the first chord symbol a of a chord transition, the *normalized conditional entropy* of the random variable *B* over all possible subsequent chords is defined as

$$\begin{array}{c} \bar{\text{H}}\left(B|A=a\right)=\text{H}\left(B|A=a\right)/\log_{2}\left(|B|\right), \end{array}$$ (3)

where the normalization factor log_2_(|*B*|) the maximum entropy attainable by a random variable on |*B*| distinct elements.

The black bars to the left of the heatmaps show these normalized conditional entropies for the 25 most frequent chords in major and minor, respectively. They indicate a certain variability between these chords with respect to their transition probabilities as was expected. In the following, we examine the relation of this variability to certain chord features such as inversions, suspensions, added notes, and applied chords. Finally, a chord may appear over pedal notes (e.g., all chords in the brackets of V[vi7 ii V7 I] occur on the pedal V). The role of these chord features in tonal harmony is illuminated by analyzing how well they predict subsequent chords.

We want to know which of the five chord features have a statistically significant effect on the predictability of the subsequent chords. For example, we expect that suspended chords should increase predictability, because the following chord is most likely a resolution of this suspension (e.g., V(64) → V). Using normalized conditional entropy $\bar{\text{H}}$ as a measure of predictability, we compare chords having a certain feature to random chord samples and perform a *one-sample bootstrap hypothesis test* [53]. The fundamental assumption of this resampling approach is that the relationship between an unknown population *X* and a sample **x** = (*x*_1_, …, *x*_*N*_)∈*X*^*N*^ of size $N\in N_{+}$ is analogous to the relationship between the sample **x** and its resamples $x^{*}=\left(x_{1}^{*},\ldots ,x_{N}^{*}\right)\in X^{N}$,

$$\begin{array}{c} X\to x\approx x\to x^{*}. \end{array}$$ (4)

In the following, the normalized conditional entropy values of all chords in the dataset are here taken as the sample **x** (separately for major and minor). Let *f* be a chord feature and *μ*(*X*) be the mean of *X*. We test whether the mean *μ*_*f*_ of normalized conditional entropies of chords having feature *f* is significantly different from the mean *μ*(**x**) of normalized conditional entropies of randomly sampled chords from the unknown population *X*. The null hypothesis *H*_0_: *μ*(**x**) = *μ*_*f*_ is tested against the alternative *H*_1_: *μ*(**x**)<*μ*_*f*_ or *μ*(**x**)>*μ*_*f*_.

The bootstrap assumption given in Eq 4 is applied in order to simulate the random sampling of **x** from *X* using bootstrap resamples x*. To implement the null hypothesis, the normalized conditional entropies *x*_1_, …, *x*_*N*_ are shifted such that their mean *μ*(**x**) equals *μ*_*f*_,

$$\begin{array}{c} {\tilde{x}}_{i}=x_{i}-\mu \left(x\right)+\mu_{f},\;\;\text{for}\;i=1,\cdots ,N. \end{array}$$ (5)

The bootstrap procedure generates a large number *B* of *bootstrap samples* ${\tilde{x}}_{j}^{*}$ (*j* = 1, …, *B*) and calculates their respective means $\mu ({\tilde{x}}_{j}^{*})$. The proportion of these bootstrap sample means that is more extreme than the actual sample statistic *μ*(**x**) determines whether *H*_0_ can be rejected with a *p* value of

$$\begin{array}{c} p=\frac{2}{B}\min(\sum_{j=1}^{B}1(\mu \left({\tilde{x}}_{j}^{*}\right)\leq \mu \left(x\right)),\sum_{j=1}^{B}1(\mu \left({\tilde{x}}_{j}^{*}\right)\geq \mu \left(x\right))), \end{array}$$ (6)

and significance level *α*, where **1** is the indicator function.

A major advantage of this method is that it does not require any specific assumptions about the distribution of **x** and the test statistic [53]. In particular, one does not have to assume that the population is normally distributed. For all subsequent analyses, the number of bootstrap resamples is *N*_*B*_ = 100, 000 and the significance level is set to *α* = .01.

**Fig 7:**
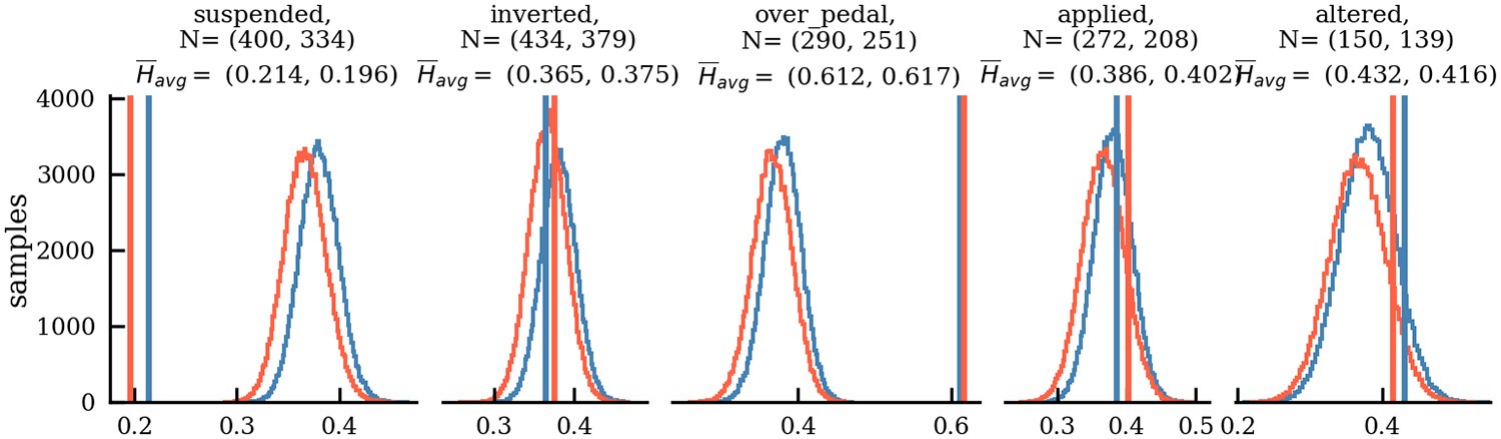
Entropies based on chord features. Average normalized conditional entropies ${\bar{\text{H}}}_{\text{avg}}$ of chord types with a certain feature (vertical lines) for major (blue) and minor (red) compared to bootstrap samples of the same size *N* (histograms) under the null hypothesis. Subfigures display the different features suspensions and added notes (“suspended”), inversions (“inverted”), chord over pedals (“over pedal”), applied chords (“applied”), and chords with altered roots (“altered”). The first number in parentheses refers to the major mode, the second number to the minor mode.

The results shown in Fig 7 reveal that chords with suspensions (left panel) and chords on top of pedal notes (central panel) are significantly different from a random chord sample in terms of their predictability of consequent chords as measured by the normalized conditional entropy. Chords with suspensions have on average a much lower entropy than non-suspended chords, which indicates that the implied voice-leading strongly increases predictability of the subsequent event. Inverted chords (second to the left panel) are not significantly different from the average chord sample. Although inversions can have strong implications (e.g., V2 → I6), they do, for instance, also occur in contexts of chord prolongation (e.g. I → I64 → I6 → I). Hence, chord inversion as a categorical feature does not significantly affect the predictability of the subsequent event. From a musicological perspective, the most surprising finding is that chords over pedal notes (central panel) are much less predictable than randomly selected chords. It suggests that the pedal note is harmonically much more important for the prediction of the next event than the chord itself. Another unexpected finding is that the average entropy of applied chords (second to the right panel) is not significantly lower than that of random chords. Although applied chords are expressed in reference to a specified scale degree (e.g. the ii in V/ii), this implied scale degree follows only in 689 of all 2,641 instances in major and only in 670 of all 2,567 instances in minor (both 20.7%). Finally, we observe a non-significant trend that chord alterations (right panel) achieved by transposing the root up or down by semitone (e.g., #vii, bII) decrease chord predictability.

As the unigram model showed, the majority of chord tokens consists of a small set of chord types. The transition probabilities from Fig 6 allow also to identify the most frequent chord bigrams. In particular, among all transitions in major, 44.9% contain variants of I and 64.9% contain a V type. 10.9% of all transitions in major proceed from a chord with root I to a chord with root V, and 14.7% move in reversed direction. In the minor mode, 23.8% of all chord transitions contain a tonic chord with root i type and 62.4% contain a V type. 5.8% of all chord transitions are from a i type to a V type, and 7.3% proceed from a V to a i type. This overabundance of chord progressions from and to variants of I, i, and V strongly advocates the privileged roles of chords on these roots in tonal harmony to create local patterns. One can also observe an asymmetric relationship between chord types I and V as well as between i and V. This points to the hypothesis that tonal harmony is largely asymmetric and therefore conveys directedness [18].

## Directedness: Asymmetry of chord progressions

According to the third main feature of tonal harmony, *directedness*, we expect to find a prevalence of asymmetric chord progressions, i.e. that the probability of the chord bigram a → b is different from that of b → a. For each mode *m* ∈ {major, minor}, the probability *p*_*m*_(a → b) is estimated by the relative frequency *count*_*m*_(a → b)/*N*_*m*_, where *N*_*m*_ is the number of segments in that mode. The dataset contains 16,187 chord transitions in the 357 major segments, and 10,979 chord transitions in the 572 minor segments. Directedness is operationalized using the *bigram symmetry*

$$\begin{array}{c} sym_{m}\left(a\to b\right)=\min\{\frac{p_{m}\left(a\to b\right)}{p_{m}\left(b\to a\right)},\frac{p_{m}\left(b\to a\right)}{p_{m}\left(a\to b\right)}\}, \end{array}$$ (7)

for non-zero values of *p*_*m*_. Chord repetitions are excluded because they are symmetrical by definition. A bigram symmetry of 1 implies *p*_*m*_(a → b) = *p*_*m*_(b → a) and hence perfect symmetry, whereas lower values indicate asymmetrical behaviour for a given pair of chords. Values greater than 1 are not possible. The overall *mode symmetry* is defined as the average bigram symmetry

$$\begin{array}{c} \bar{sym}\left(m\right)=\sum_{a}\sum_{b}p_{m}\left(a\to b\right)\cdot sym_{m}\left(a\to b\right), \end{array}$$ (8)

where a and b are arbitrary chord types such that a ≠ b, and both a → b and b → a are bigrams in mode *m*.

**Fig 8:**
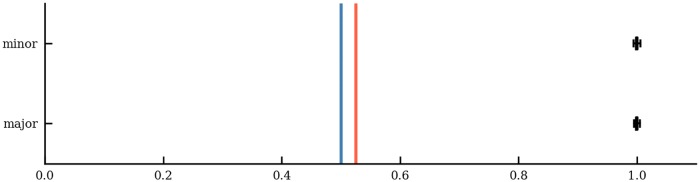
Mode symmetries. Mode symmetries for major and minor (blue and red vertical lines) and bootstrap resamples under the null hypothesis that chord progressions are symmetrical (histograms).

Fig 8 shows the mode symmetries for major (blue line) and minor (red line). The boxes show the bootstrapped mode symmetries under the null hypothesis that chord transitions are symmetrical. Because of the vanishingly small variances, one can deduce that the observed mode symmetries are highly unlikely under the symmetry assumption. This corroborates the fact that harmonic progressions are significantly asymmetrical. In fact, progressions a → b are approximately twice as common as the reversal b → a ($\bar{sym}\left(\text{major}\right)=.5$, $\bar{sym}\left(\text{minor}\right)=.53$), under the assumption that a → b is more frequent than b → a. Simply put, tonal music would substantially change its character when played backwards.

**Fig 9:**
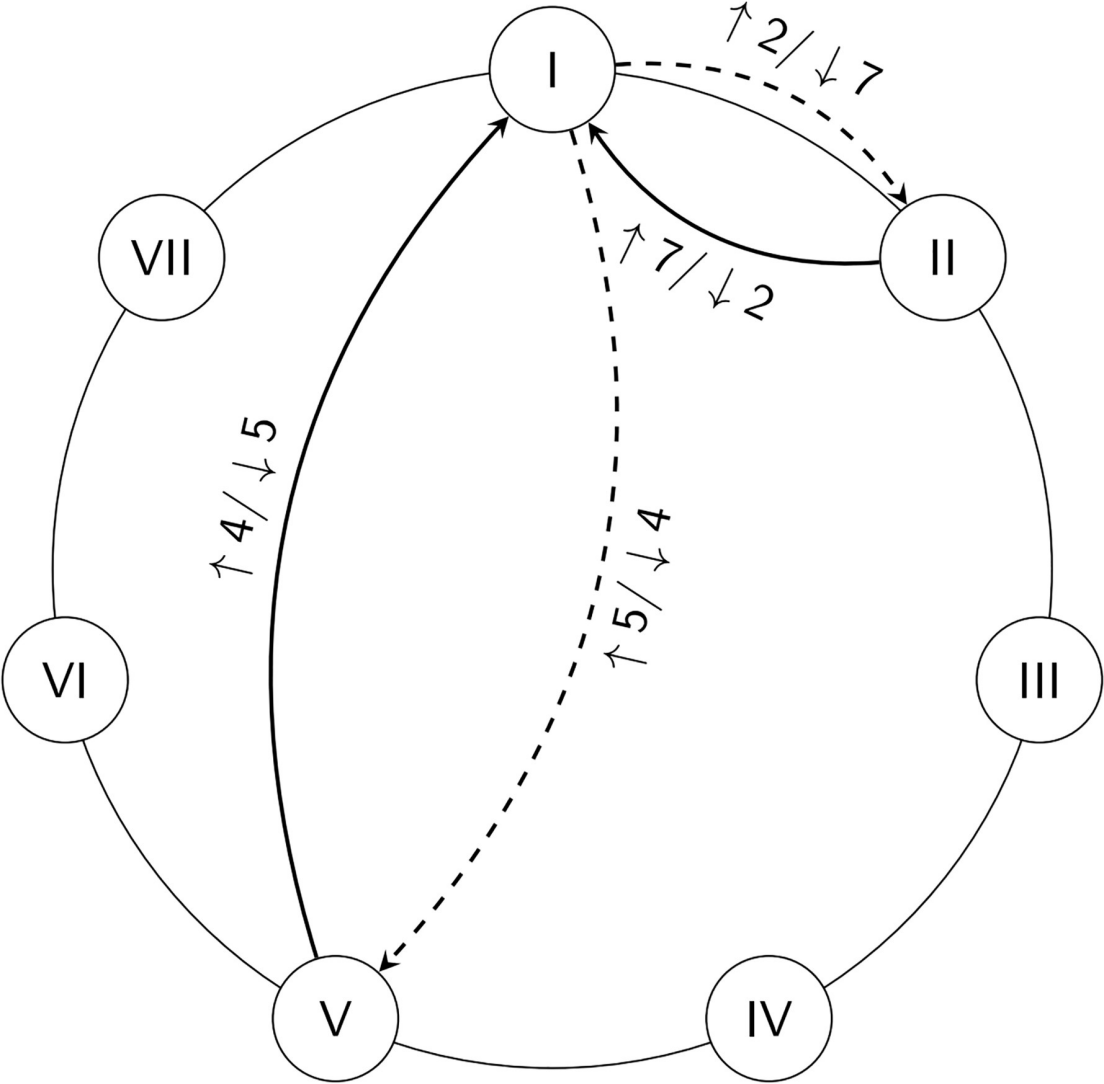
Examples of chord root progressions. Authentic root progressions are shown as solid arrows and plagal root progressions are shown as dashed arrows. Note that the example shows progressions involving I.

Chord transitions can further be characterized in terms of the size and the direction of the interval between the respective roots. Root progressions are traditionally categorized as either *authentic* or *plagal*. Table 1 lists all possible root progressions and Fig 9 shows four examples of root progressions: authentic progressions (solid arrows) from V to I and from II to I, and plagal progressions (dashed arrows) in the reverse direction. Note that we refer to generic (rather than specific) intervals here, i.e., we do not distinguish between major and minor intervals (as in the case of seconds and thirds and their complementary intervals).

**Table 1:** Root progression intervals.

symbol	interval	complement	type
1	unison	octave	stationary
↑2	ascending second	descending seventh	authentic
↓2	descending second	ascending seventh	plagal
↑3	ascending third	descending sixth	plagal
↓3	descending third	ascending sixth	authentic
↑5	ascending fifth	descending fourth	plagal
↓5	descending fifth	ascending fourth	authentic

**Fig 10:**
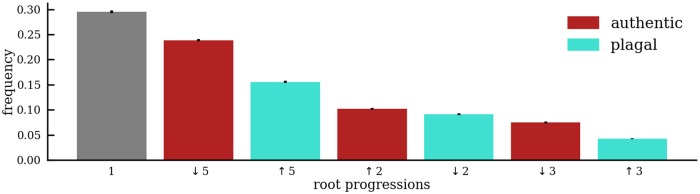
Chord root progressions in the ABC. Distribution of bootstrapped means of root progression frequencies between chords in tonal harmony. Error bars show the standard deviation from the mean. Authentic progressions are more common than plagal progressions, and the ranked interval sizes of root motions are fifths, seconds, and thirds.

Authentic motions are considered to be prevalent in tonal harmony [36, 37], comprising descending odd-numbered intervals (thirds, fifths, sevenths) and ascending even-numbered intervals (sixths, fourths, seconds). Plagal progressions reverse the direction of the authentic intervals. The statistical prevalence of authentic progressions is evident in Fig 10.

The data contains 41.6% authentic progressions (descending fifths: 23.9%; descending thirds: 7.5%; ascending second: 10.2%) and 28.9% plagal progressions (ascending fifths: 15.6%; ascending thirds: 4.2%; descending seconds: 9.1%). Almost one third of all chord transitions (29.5%) maintain the same root and do not constitute any harmonic progression at all. These can, for instance, be attributed to chord resolutions of suspensions or chord arpeggiations where the bass note changes but the chordal root stays the same. The analysis shows that tonal harmony favors authentic progressions over plagal ones and thus differs from Rock/Pop tonality, which shows the reverse pattern [38]. In particular, tonal harmony evinces a preference for fifth-related progressions in both directions, a trend that emerged over the course of the 16th- and 17th centuries [20]. The clear preference for authentic (i.e. descending) fifth-related authentic progressions is shared with Jazz [54], which conveys a similar directedness in time.

## Hierarchy: Relationship between chords and keys

**Fig 11:**
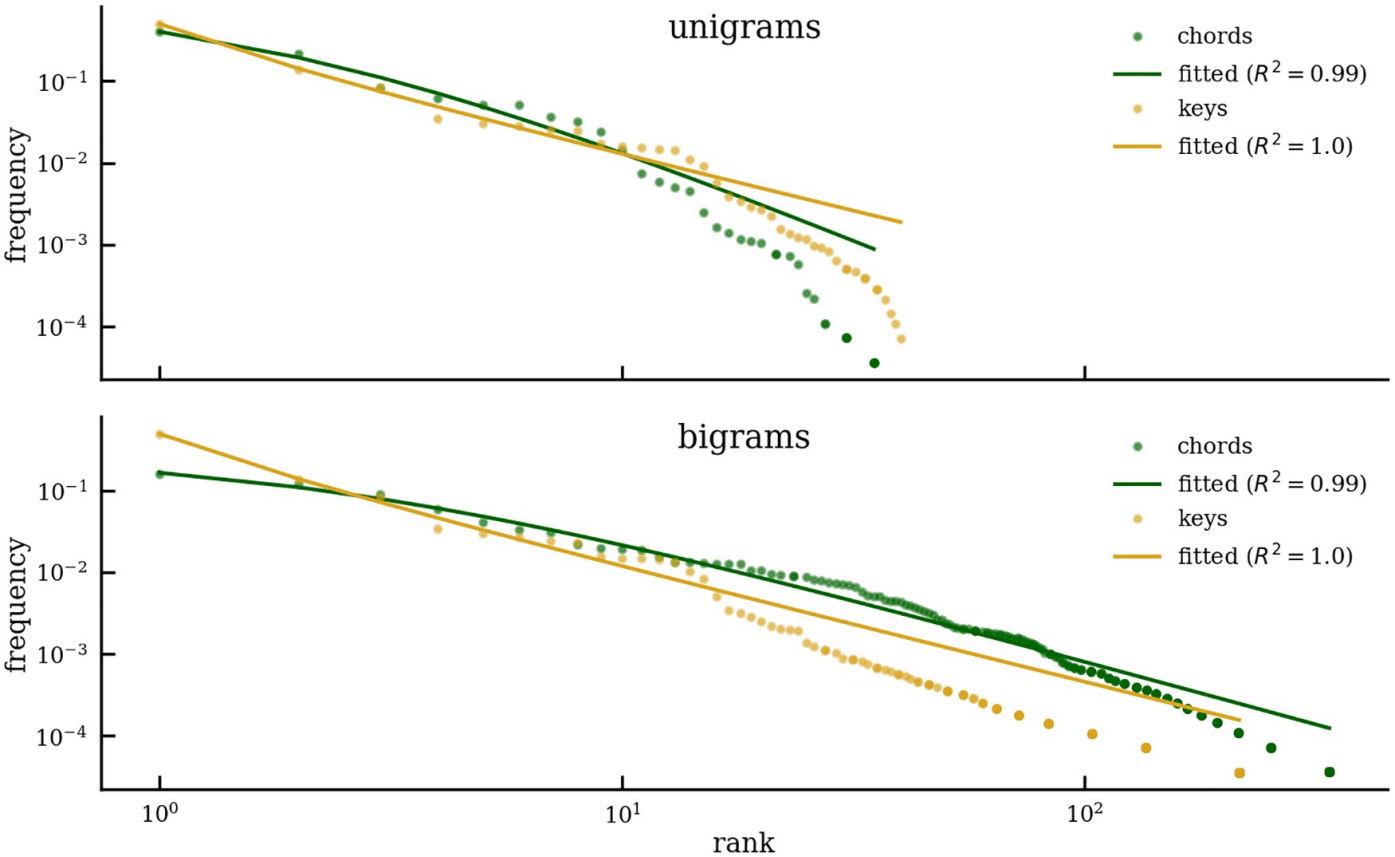
Chords vs. keys. Best fit of Zipf-Mandelbrot function to unigram (top) and bigram (bottom) rank vs. frequency curves as indicated by the coefficient of determination *R*^2^.

The hierarchical organization of tonal harmony is assumed to be another of its defining features. The encoding of tonal harmony in the corpus allows for the comparison of two hierarchical levels, namely chords and keys, and for the assessment of the degree of similarity between them. A comparison of these levels is only sensible if the symbols are based on the same lexicon. Since the key lexicon is the same as the lexicon of chord roots (both are expressed by Roman numerals), one can compare the distributions of key and chord root unigrams, as well as their respective bigrams. Comparing the rank-frequency relations in keys and chords (Fig 11) indicates that the shape of both distributions is very similar for both unigrams (top) and bigrams (bottom), meaning that these two hierarchical levels share the property that few items account for large proportions of the probability mass, whereas many items occur rarely, often only once. Although the fitted curves only poorly model the tails of the distributions, a power-law-like trend can be observed (for the interpretation of the accuracy measure *R*^2^ one may refer to section “Centricity: Structure of the Chord Lexixon”).

To investigate whether or not the chord and key transitions are significantly different, we apply a two-sample bootstrap test [53]. This test is analogous to the procedure described above (section “Referentiality: Chord Transitions”), the difference being that both the chord and the key transitions are resampled.

We interpret the transition tables for chords and keys as vectors of dimensionality equal to the squared lexicon size and calculate their cosine distance. The null hypothesis states that this cosine distance is equal to zero, meaning that chord and key transitions are identical. To implement this hypothesis, we generate bootstrap resamples from the combined list of chord and key bigrams. Each resample is then split according to the proportions of chord and key transitions. The mean and standard deviation of the resampled distance distribution are *μ* = 2.6 × 10^−4^ and *σ*^2^ = 9.58 × 10^−5^, respectively. The cosine distance between chord and key transitions of the original sample is.64 and thus extremely unlikely under the null hypothesis. One can conclude that transitions at the two hierarchical levels of chords and keys follow a similar shape but differ with respect to the concrete transitions. This finding renders it unlikely that both hierarchical levels conform to the identical set of rules, thus underpinning musicological positions critiquing assumptions of hierarchical uniformity [10, 55].

## Conclusions

This article presents a empirical characterization of tonal harmony, the core organization system of Western music. Using the *Annotated Beethoven Corpus*, one of the largest datasets of expert-annotated harmonic analyses available to date, we adopt a statistical approach to model tonal harmony and to advance the methodological state-of-the-art in music research. We propose an overarching model employing four core dimensions (centricity, referentiality, directedness, and hierarchy) and explore the dataset under that paradigm. Importantly, we do not claim that these dimensions provide an exhaustive characterization of tonal music, as we leave other structural aspects such as meter, rhythm, voice-leading, and hierarchical syntax out of account. Nonetheless, the dimensions considered here constitute central pillars of the Western musical system.

Our results have also cognitive implications and may provide a resource for the modeling of the competence of listeners who acquired the rules of tonal harmony through statistical learning [17, 56]. Tonal harmony exhibits communicative efficiency through a small number of highly frequent elements; it provides listeners with chord features as cognitive markers enhancing the predictability of subsequent musical events; it uses chord progressions to convey directedness in time; and it communicates differences between structural levels by treating chord and key transitions differently.

The four model dimensions may also provide a core for the empirical characterization of other musical cultures in the world and other musical features than harmony. Moreover, Western musical systems prior to or after the common-practice period can be illuminated by comparing them along the proposed axes. Our model might further prove beneficial for the comparison of common-practice tonal harmony with modern musical systems such as Rock, Pop, or Jazz. Finally, this study is conceived as complementary to more traditional avenues in music research, bridging empirical methods and musicological theorizing by providing the statistical foundations of tonal harmony.
